# Medicine and Surgery in Mesopotamia

**Published:** 1916-12

**Authors:** Carey F. Coombs

**Affiliations:** Assistant Physician, Bristol General Hospital


### MEDICINE AND SURGERY IN MESOPOTAMIA.

BY

CAPT. CAREY F. COOMBS, R.A.M.C. (T.).

*Assistant Physician, Bristol General Hospital.*

I PUT medicine first, not because of any personal predilections, but because during my short stay in Mesopotamia the surgical cases were "snowed under" by cases of sickness. Not only so, but most surgical cases presented evidence sooner or later of non-surgical disease. It may be well to look first in a general way at the principal causes of this heavy incidence of sickness and disease.

First of all comes the heat. When I reached the 23rd Stationary Hospital at Amara, to which I wa[illegible] attached, the hot weather was just beginning. To very many of the troops tropical heat was an entirely new experience, living under canvas as the vast majority did. Moreover, there is something peculiarly potent about the heat in Mesopotamia; even those who were accustomed to higher temperatures, in India and elsewhere, found it very trying. The effect of the sun as a factor in causing disease showed itself chiefly in a lowering of resistance to infection. Everyone lost weight, whether sick or not, and this was, I believe, in large measure due to the climate. Of actual heat-stroke I saw none myself, but I heard of a few cases even as early as June. In 1915 I am told there was a good deal. Patients in thin hospital tents suffered much from the heat.

Another potent cause of disease was the water supply. All water was drawn from the Tigris. The habits of the Arabs, and their apparent freedom from water-borne disease, furnished a striking illustration of acquired immunity. Within a comparatively short stretch of river-bank you saw people washing clothes, defecating, bathing, and drinking the river water "neat." Add to this picture an occasional corpse, and the pathogenic properties of Tigris water are not hard to imagine. Apart from the bacterial content of the water, however, it seems that the sand in suspension and certain salts in solution acted as intestinal irritants. Scarcely anyone escaped a sharp attack of diarrhœa as an early experience of Mesopotamia's hospitality. Added to these natural properties were all sorts and conditions of intestinal bacteria, from the cholera vibrio downwards. Against these various insults to the alimentary canal the Arab has been immunised from his infancy up by a very simple method—that of drinking the water in increasing doses. The dosage being regulated by the size of the hand that scooped the water up out of the river it follows that the Arab is educated, by doses increasing in a true proportion with his increasing body weight, to swallow filth. Probably this means a high infant mortality, but at least the survivors are adequately immunised.

In most places the water was collected into tanks, sedimented by gravity, and sometimes also by the addition of alum, and the fluid thus cleared was further disinfected with bleaching powder. Some of us boiled a private supply for ourselves, but it was impossible to practise this on the large scale. There were no receptacles for doing so, and fuel was scarce.

A third factor in the production of disease was a plentiful supply of biting insects. The chief of these were the mosquito, the sand-fly, and a peculiarly annoying fly like the English house-fly, which bit, and painfully, through thick socks, trench stockings, and so on. I did not see many lice, but in the trenches they were plentiful, I am told. Not only did these insects spread disease by their bites, but also by their passage from fæces to food. It was quite impossible to keep one's food free from them, and they must have been a potent factor in the spread of the intestinal infections.

Another thing which certainly predisposed to disease, as well as causing it directly in a few instances, was the lack of food. I heard of beri-beri in real outbreaks, but I saw only one or two cases that might have been so diagnosed. Scurvy also occurred, though not within my own observation. But the way in which the food shortage did operate was in lowering resistance to infection, and especially to the intestinal infections. The Red Cross Society did good work in bringing us supplies of various invalid foods, but without these it was hard to treat patients suffering from various types and degrees of diarrhœa. The bread was rather sour, and there was practically no fresh milk. Chicken were to be had; though small, they made good eating. Also there were fish in the river, but of a muddy and not alluring flavour.

Finally, I am sure a place in the production of and predisposition to disease must be given to the depressing mental outlook. The country is a vast, colourless plain, almost devoid of trees, shimmering in the heat. The campaign was not prosperous, and we were cut off from the comforts of home and civilisation. The result was a depression from which there was nothing external to distract the mind. Among the Indian troops home-sickness was so potent that all kinds of devices, some of them most desperate, were resorted to in order to compass an escape from the country. The British soldier, I am convinced, felt it even more severely, but, as is his wont, said nothing about it.

Put all these factors together, and it is not surprising that there was much disease. Indeed, it is marvellous that anyone got through, especially among the unseasoned troops. The next point to note is the chief features of the diseases met with.

First, there were the various degrees of diarrhœa. Much of this, I think, was to be ascribed to the water supply. Almost everyone on first arriving in the country showed more or less evidence of intestinal irritation. This varied from a mild relaxation of the stools to an intense, almost choleraic diarrhœa, but it was usually a febrile. The worst feature of it was the weariness and susceptibility to infection which followed. Next above these "Tigris" diarrhœas came the cases of febrile enteritis and colitis. In cases of this type the fever lasted for two days up to ten days. It seldom ran high. The diarrhœa showed an annoying tendency to persistence with relapse. Specific types of diarrhœa, such as amœbic dysentery, were apparently rare.

On the other hand, it was difficult to draw a line of demarcation between cases of this type and cases of the enteric group of infections. Of this more presently. Worst of all were the cases of cholera. Some doubt rested as to the true specificity of these cases. Most of them were said to be milder than ordinary Asiatic cholera. Three cases that I saw—about the first of their kind to turn up at Amara—all proved fatal, one in a few hours, the others after several days, and in spite of determined efforts with hypertonic saline. Even in the severest of these, however, the stools failed to yield the characteristic vibrio. There was some suspicion that these might have been some form of ptomaine poisoning, but so far as we could trace it the evidence was against this theory. In the final stage of these cases I was struck by the air-hunger type of respiration similar to that seen in acidosis.

Second, there was a remarkable medley of cases of pyrexia. From the general muddle several types stood out more or less distinctly. There was a short fever which we called "Mesopotamia fever." I think it was identical with the short fevers of other tropical countries. It seemed to be due to the heat, possibly acting as a factor predisposing the body to infection by feeble organisms which would otherwise make little or no impression on it. It affected those who had newly come to the country. The onset was abrupt, with a sharp rise of temperature, severe headache, bounding, rapid pulse, face flushed, expression weary, eyes injected and tongue furred. The whole thing passed off in forty-eight hours or so. Second, there were the enteric fevers, probably including paratyphoid. In the absence of proper laboratory arrangements not much could be done towards investigation of these. Yet there is little doubt in my mind that some of the febrile diarrhœa to which I have alluded above represented "modified" types of enteric infection. We may suppose either that there is a much larger number of pathogenic strains belonging to the typhosus-paratyphosus group of organisms, or that inoculation, particularly with the mixed vaccines, has produced modified types of the reaction to infection. The signs which appeared to be of most service in distinguishing between the enteric cases and those which resembled them were the tumid abdomen with loss of abdominal reflexes, the rose spots, the dicrotic pulse slow in proportion to the degree of fever, and enlargement of the spleen when it was present. We had one case of perforation, in which my colleague, Lieut. T. M. Body, R.A.M.C., discovered and closed the perforation with what would have been signal success under happier circumstances. But the man was too exhausted by the climate and the long trip down river, and he failed to get through. Then there were the malarial fevers. These were not so numerous as I had expected, but we had a few cases of benign tertian infection, and quite a definite group of cases which, judging from their favourable reaction to vigorous quinine treatment, were examples of irregular malarial infection. In these the fever was continued with a tendency to periodic exacerbations at intervals of two or three days. Somewhat similar to these were cases, of which my own was an example, of fever returning in short paroxysms at intervals of five or six days, and characterised by pains in the legs, loss of weight, and anæmia. This seems to me to be identical with the severer types of "trench fever" described in Flanders, in the Russian armies in Volhynia, and elsewhere. But apart from these more or less definite types of fevers there were all sorts and kinds of continued pyrexia to which it was impossible to affix any label. These offered a rich opening for research, tantalising because the means of achieving it were not within reach. One thing may be remarked as to the general nature of the fever, and that is that in all types the febrile reaction seemed to be exaggerated out of proportion to the provoking cause.

Very much might be written about the evidences of general exhaustion. These are, of course, seen in every army. They constitute a most interesting subject, but here it would be out of place to do more than indicate the features peculiar to Mesopotamia. Loss of flesh was common to everyone. Those who had diarrhœa or some infection lost weight faster than they would have done in a more familiar climate, while those whose health was comparatively good nearly all got thinner. The most intense cases of pure exhaustion that I have ever seen were among men who had been engaged in the fruitless attempt to relieve Kut. One case in particular struck my imagination. The patient was a young artilleryman of powerful physique, a collier in civil life. Ordinarily he would strike one as a thick-skinned son of toil, unimpressionable in the least degree. Yet he lay curled up in bed like a meningitis case, with the bed-clothes drawn over his head, weeping quietly. His legs were very hyperæsthetic, but there was no organic lesion. He became extremely constipated, and his abdomen blew up hard to such an extent that one of my colleagues, who saw him when I was ill, thought he would need laparotomy. However, an enema succeeded, and from his subsequent course it became clear that the whole thing was functional, and due in some obscure way to the wear and tear to which he had been subjected. Nightmare was common. Many of the men wore an extremely anxious look. This was noticeable even among the slighter cases of sickness. Pains in the legs were common, and seemed to me to be caused in some way by exposure and fatigue. The "soldier's heart" was as frequently encountered as among any of the armies, with the possible exception of the men from Gallipoli.

One very striking feature, observable in all the various types of diarrhœa and also of fever, was the impossibility of convalescence. No one ever picked up strength as well as might have been expected. Particularly was this noticeable in men of forty or over. The wastage due to sickness was greatly aggravated by it. As one of my friends put it: "If you get your head below water in this country you don't get it up again."

Of surgical work I can say but little. When we arrived the heavy casualty lists that marked the attempts to relieve Townshend's army in Kut were dwindling. The one striking feature of the cases I saw was the absence of serious wound infection. That this was due to the nature of the soil seems probable, for there was quite a strong tendency to sepsis in small lesions such as insect bites on the legs. No case of tetanus came under my own notice. Recovery from serious wounds must have been grievously handicapped by the difficulty of getting the men out of the fighting line and back to the hospitals.

One must recognise the immense difficulties of organising a medical service on the large scale that was necessary. It is over 6,000 miles from England to Basra even by the most direct route, and Basra is 1,600 miles from Bombay, the nearest available centre of civilisation. Amara was about 150 miles above Basra, and the front was 100 miles farther on. The only means of transport was by the Tigris, a very difficult river to navigate; there was no railway, and no road available for motor transport.

It would be unfair to conclude this paper without saying a word as to the splendid work of the R.A.M.C. Never did the motto of the Corps, "*In arduis fidelis,*" find a more signal fulfilment. When we arrived the personnel was far below the minimum of what was necessary. During my short stay many fresh officers arrived, and this redressed the disproportion ; but those who had borne the heat and burden of the earlier part of the campaign, particularly of the period following Ctesiphon, and including the attempts to relieve Kut, had accomplished wonders of endurance and perseverance. This applies to the I.M.S. as well as to the home Corps.

Various Bristol men were at work in Mesopotamia. At Amara practically all the bacteriology was in the hands of Major F. P. Mackie, I.M.S., who accomplished the apparently impossible by working through the heat of the afternoon day after day. Captain Green-Armytage, I.M.S., was also there, attached to the Commander-in-Chief's Staff, but I did not happen to see him. Captain A. N. Thomas, also of the I.M.S., was invalided before I got out. Many whom I met counted themselves among the friends of Frank Shingleton Smith, who fell at Ctesiphon. The work of Physician to No. 32 General Hospital was being carried on by Lieut. F. G. Thomson, R.A.M.C., of Bath, when I last heard. Outside the medical services there were many West Country units serving in Mesopotamia, and it was pleasant to hear the familiar speech of Wessex in the hospital tents of Amara.

